# Peer Review of “Impact of Modifiable Risk Factors on the Occurrence of Cutaneous Leishmaniasis in Diyala, Iraq: Case-Control Study”

**DOI:** 10.2196/31513

**Published:** 2021-07-30

**Authors:** Riham Al-Dubaiee

**Affiliations:** 1 Ministry of Public Health and Population Sana'a Yemen

**Keywords:** cutaneous leishmaniasis, outbreak, Iraq, risk factors, risk, disease, infectious disease, disease prevention, prevention

*This is a peer-review report submitted for the paper “Impact of Modifiable Risk Factors on the Occurrence of Cutaneous Leishmaniasis in Diyala, Iraq: Case-Control Study.”*

## Round 1 Review

### General Comments

This paper [1] is very useful, as cutaneous leishmaniasis is a neglected infectious disease in the Eastern Mediterranean region.

### Specific Comments

#### Major Comments

1. What is the accurate definition of the controls, mentioned as family members? If they are family members, could there be risk factors in most of the housing characteristics that they share, such as animals in the house, electricity, and distance from animals?

#### Minor Comments

2. In the *Background* section, I suggest putting the epidemiology globally, followed by the Eastern Mediterranean region, then Iraq (was interrupted).

3. I have a comment on mentioning the risk factors in the *Background* section.

4. In the *Results* section, “usage of fogging and bed nets” is repeated twice in two paragraphs.

5. In the *Methods* section, the ratio of cases:controls should be mentioned.

6. Some of the references need to be revised.
